# Dementia and Imagination: a mixed-methods protocol for arts and science research

**DOI:** 10.1136/bmjopen-2016-011634

**Published:** 2016-11-02

**Authors:** Gill Windle, Andrew Newman, Vanessa Burholt, Bob Woods, Dave O'Brien, Michael Baber, Barry Hounsome, Clive Parkinson, Victoria Tischler

**Affiliations:** 1 Dementia Services Development Centre, School of Healthcare Sciences, Bangor University, Bangor, UK; 2 International Centre for Media, Culture and Heritage, School of Arts and Cultures, Newcastle University, Newcastle upon Tyne, UK; 3 Department of Gerontology, Centre for Innovative Ageing, College of Human and Health Sciences, Swansea University, Swansea, UK; 4 Cultural Policy, Institute for Creative and Cultural Entrepreneurship, Goldsmiths University, London, UK; 5 Age Watch, London, UK; 6 Biostatistics Department, Institute of Psychiatry, Psychology and Neuroscience, Kings College London, London, UK; 7 Manchester Metropolitan University, Manchester, UK; 8 University of Nottingham, Nottingham, UK

**Keywords:** quality of life, mixed-methods, multi-disciplinary, arts and health

## Abstract

**Introduction:**

Dementia and Imagination is a multidisciplinary research collaboration bringing together arts and science to address current evidence limitations around the benefits of visual art activities in dementia care. The research questions ask: Can art improve quality of life and well-being? If it does make a difference, how does it do this—and why? Does it have wider social and community benefits?

**Methods and analysis:**

This mixed-methods study recruits participants from residential care homes, National Health Service (NHS) wards and communities in England and Wales. A visual art intervention is developed and delivered as 1×2-hour weekly group session for 3 months in care and community settings to N=100 people living with dementia. Quantitative and qualitative data are collected at 3 time points to examine the impact on their quality of life, and the perceptions of those who care for them (N=100 family and professional carers). Repeated-measures systematic observations of well-being are obtained during the intervention (intervention vs control condition). The health economics component conducts a social return on investment evaluation of the intervention. Qualitative data are collected at 3 time points (n=35 carers/staff and n=35 people living with dementia) to explore changes in social connectedness. Self-reported outcomes of the intervention delivery are obtained (n=100). Focus groups with intervention participants (n=40) explore perceptions of impact. Social network analysis of quantitative and qualitative data from arts and healthcare professionals (N=100) examines changes in perceptions and practice.

**Ethics and dissemination:**

The study is approved by North Wales Research Ethics Committee—West. A range of activities will share the research findings, including international and national academic conferences, quarterly newsletters and the project website. Public engagement projects will target a broad range of stakeholders. Policy and practice summaries will be developed. The visual art intervention protocol will be developed as a freely available practitioners guide.

**Strengths and limitations of this study**

- Dementia and Imagination is the largest arts and dementia research study in the UK.
- The development and delivery of the research involves partnerships between universities, community arts organisations, galleries, the National Health Service (NHS) and charities.
- It combines methods and practice from health and social sciences together with the arts and humanities to address a key societal challenge—supporting the quality of life (QoL) of the growing number of people living with dementia.
- A limitation is that this mixed-methods study design cannot focus on a more robust test of effectiveness (eg, as a randomised controlled trial would) as this was beyond the available resources.

## Introduction

### Background

There is no cure for dementia, therefore attempts to maintain QoL and well-being for those living with memory difficulties are crucial. Dementia is now firmly on the international public and policy agenda, bringing opportunities for change on a wider scale for those living with the condition^1^ and the communities in which they live. In the UK, changing awareness and understanding about dementia and enabling people to ‘live well’ is central to the National Dementia Strategy.^2^ Creating dementia supportive or dementia friendly communities is a major policy objective and a societal imperative noted in the National Dementia Vision for Wales^3^ and the Prime Minister's Challenge on Dementia.^4^

There is substantial interest in the role of the arts in dementia care, largely driven by innovative practice. Arts-based activities include dance, music, theatre, creative writing, visual art and singing. Reviews of arts and dementia research suggest a range of positive benefits to those taking part, such as improvements in well-being, QoL, cognitive function and communication.^5–10^ However, associated research in this area has not kept pace, limiting the extent to which science informs practice and service improvement. All the reviews highlight methodological issues, which limit the impact of findings.^5–10^ In particular, most of the published research around visual art programmes for people living with dementia have used small or opportunist samples of people. Belfiore and Bennett^11^ argue the rhetoric around the cultural, social and health benefits of arts is often derived from unquestioned, deep-seated beliefs about the role and functions of the arts in society which have become widely accepted. Nevertheless, longitudinal population studies suggest that cultural activities can have a positive impact on health.^12^

### Summary of current study

The Dementia and Imagination research is a multidisciplinary collaboration, bringing together arts and science to address current evidence limitations. The development and delivery of the research involves partnerships between universities, community arts organisations, galleries, the National Health Service (NHS) and charities. The research combines methods and practice from health and social sciences together with the arts and humanities to address a key societal challenge—supporting the QoL of the growing number of people living with dementia. It is a large programme of work, using the medium of visual art as an exemplar and catalyst for change. It examines the beneficial impact of taking part in art activity on the QoL of people living with dementia, together with the cost-benefit, and the perceptions of those who care for the participants living with dementia. More broadly, through public engagement and research dissemination activities it uses artistic output from the study, and artistic interpretations of the data and methods, together with quantitative and qualitative data to challenge the public perception of dementia. It examines processes of delivering the intervention and outcomes to advance understanding and be of value to research, policy and practice. Online supplementary appendix 1 presents the different components of the study, set out as work packages. This paper describes the data collection methods and analyses for the different groups of participants. The research started in July 2013, and is due to complete February 2017.

10.1136/bmjopen-2016-011634.supp1 supplementary appendix

### Rationale

As previously noted, reviews of arts activities suggest a number of positive benefits. There is currently no evidence to suggest that one art form is more appropriate or beneficial than another in terms of psychosocial outcomes for people living with dementia. This research focuses on visual art activities as a starting point in expanding the evidence base. It builds on small scale but appropriately conducted exploratory studies delivered in galleries, museums and care settings, which suggested some positive outcomes for people living with dementia taking part in visual art programmes.^13–15^ From a theoretical perspective, visual art programmes may be beneficial for people living with dementia. It is suggested that the experience of visual art-based participation can provide marginalised people creative ways to represent their experiences and extend communication and facilitate social interaction.^13–15^ Visual art forms such as pictures, photos and sculptures endure and can be used in a variety of situations or exhibitions to demonstrate that people with dementia can accomplish and learn new things, despite limited mobility or cognition.^16^ In turn, the artefact can provide cognitive stimulation. However, with the exception of a qualitative study of a gallery-based intervention,^i^^17^ there has been little research or theorising about the underlying theoretical mechanisms through which benefits develop and are sustained.^6^ ^18^ Consequently, the theoretical basis underlying efficacy is investigated in this research. This informs the development of the visual arts intervention, delivered in three settings (community venues, residential care and NHS inpatient wards) and evaluated at three points in time.

Many people with dementia wish to participate in activities, feel valued and have a sense of purpose.^19^ They wish to remain within their communities, in the home of their choice, near their family, carers and friends.^20^ In other words, they wish to remain socially connected. Socially inclusive societies are important for the QoL of people living with dementia.^3^ ^4^ However, the social representation of dementia often has a stigmatising effect on everyday interactions for both the person with dementia^21^ and their carer or partner. The general public often has a narrow and negative way of conceptualising dementia.^22^ In the face of this adversity, people with dementia (and their carers) can become disconnected, marginalised and excluded from society. Many people with dementia have minimal or no access to good quality intellectual or sensory activities, and those that do exist are often much below the person's level of functioning.^14^ This research examines the disconnection commonly experienced in dementia, and changes in social connectivity that can be facilitated by a stimulating visual arts intervention.

The stereotypes and stigma associated with dementia as previously described contributes to creating a pervasive, negative attitude known as excess disability, the tendency to attribute more disease or condition-associated impairment than the actual capabilities of the person with dementia.^23^ However, social networks have the potential for influencing behaviour and attitudes and this effect is described as social contagion.^24^ Thus, further aims of this research are to (1) use social network analysis to understand the social contagion through the implementation of good practice throughout the artists and practitioners involved in the project and beyond and (2) examine changes in perceptions and beliefs about people with dementia in the social networks of artists and professional carers of people with dementia.

The evidence review by the Mental Health Foundation^8^ noted that the community impact of arts activities has received little attention; however, activities have the potential to break down stereotypes, reduce stigmatising behaviour, foster social cohesion and raise awareness within the wider community. Creative outputs have the potential to counter stereotypes that often assume that people with dementia are unable to express their individuality in a way that is understood by others.^25^ Visual outputs can be used in creative ways to engage the public about health-related issues.^26^ Thus, public engagement activities will be a key focus in the final stages of the research.

The research programme addresses the following objectives:

- To develop a theoretically informed visual arts intervention;
- To examine the impact of the visual arts intervention on the social connectedness, QoL and well-being of people living with dementia;
- To examine family and professional carers attitudes towards dementia, and whether they change through their exposure to the visual arts intervention;
- To examine the cost–benefit of the visual arts intervention;
- To explore qualitative changes in social connectedness and communication at the microlevel between the participant and formal or informal carer(s) and other network members;
- To undertake public engagement activities, challenging perceptions of dementia;
- To understand the implementation of arts and health research, exploring the extent to which ‘messages of change’ spread through professional networks—social contagion;
- To maximise the impact of the study for policy, practice and further research.

## Methods and analysis

### The visual arts intervention

This research builds on art viewing and making programmes for people with dementia developed by some major art galleries (eg, Museum of Modern Art, New York; the Royal Academy, London; Dulwich Picture Gallery, London; National Gallery of Australia, Canberra; and Nottingham Contemporary). These take on a variety of forms of art viewing and art making, such as exploring the art history of an artwork and at other times using it as the starting point for creative discussion. They usually involve small groups of people meeting weekly.

### Intervention development

Visual art programmes are by their nature ‘complex’ in that they contain several independent interacting components (eg, settings, people receiving and delivering, their behaviours and responses, etc) which create difficulty in understanding what the ‘active ingredients’ are that bring about changes in outcomes.^27^ Complex interventions are often implemented in a diverse manner by people with variations in skills and ability, to different populations in different settings, which can influence the outcome of the intervention.^28^

The intervention development phase aims to uncover the ‘active ingredients’ to help understand how visual art interventions might be effective. This involves three approaches to theory development: (1) a realist synthesis, (2) workshops with artists, and (3) a survey of arts organisations, people with dementia and their carers. (1) A realist synthesis of published research enables an understanding of how and why something might be effective. It is used for theory development and to facilitate implementation into practice.^28^ The research team will use this approach to inform the development of the arts intervention. To the best of our knowledge, this novel approach has not previously been undertaken. The synthesis protocol is published and publicly available.^29^ (2) Workshops with stakeholders and artists (up to 40) will explore their practice to understand ‘what works’ from their perspectives. (3) A survey of organisations delivering arts programmes, people with dementia and their carers who have previously participated in similar activities, to explore what they feel worked best, and why and what was not good practice. An overarching synthesis of these three phases will produce a conceptual model to inform the visual arts intervention and a practitioners' manual (best practice guidelines for delivering the visual arts intervention), which will be finalised and publically available after the data analysis. Key aspects of the practitioners' manual form the basis of a fidelity checklist. Artists' practice is assessed from video analysis of the intervention at the beginning and end of the programme.

It is anticipated that the intervention activities will be participative and the emphasis will be on providing a stimulating, high-quality experience for the participants that require no prior knowledge or experience/skills. It will aim to encourage creativity without overwhelming people with complex instructions, be interesting and challenging, but not requiring advance artistic skills. It will encompass meaningful engagement that can stimulate imagination and discussion, not lectures or the generation of factual exchanges that rely on memory for names and dates. It will provide some structure, but create the opportunity for individual expression in a failure-free environment. For some activities, we will engage carers and staff alongside the participants.

### Study sites

There are three study sites with distinct settings: site 1, residential care facilities in the North East of England; site 2, NHS hospital wards in Derbyshire; site 3, community venues in North Wales. These settings provide different cultural contexts (eg, different industrial histories, languages and organisational practices, urban and rural contrasts) and different residential and employment locations of the participants.

### Intervention delivery

In each of the three study sites, the community arts partners (Equal Arts, Nottingham Contemporary and Denbigh County Council Community Arts) will recruit artists or use existing artists and lead the delivery of the intervention programme. These key partners have been involved in the codesign of this research from its inception, and have considerable experience of running art programmes for people with dementia and their carers. All artists will attend training provided by the research team, covering research procedures, ethics and governance. In each data collection site, the programme is delivered in a group format (estimate up to 12 people per group) as four staggered waves of 3 months duration each. The participants within each area will attend one group session a week of up to 2 hours duration.

### Participants

#### Sample size

We will recruit up to 100 participants with dementia and 100 staff and carers across three data collection sites. Attrition is expected with dementia research. To counter this possibility, we will aim to over-recruit by up to 25%. It is estimated that a 95% confidence level with 5% margin of error would require N=80. We recognise that if we were running a clinical trial of effectiveness, then the number of people with dementia required would be much larger. Numbers are chosen pragmatically, reflecting the feasibility of recruitment rates within the timescale and resources of the research programme. However, this work is the first step towards a more definitive study. For a psychosocial intervention that is not a full clinical trial (as is this arts programme), this is a relatively large number of people with dementia and yields useful process data for a future, definitive study.

### Study sample 1: people living with dementia

#### Recruitment procedures

Across all sites, a simple leaflet and information sheet is given to the participants and their families/carers outlining the study, and posters distributed where possible. The information sheet describes the project, their right to withdrawal at any stage and that any care received is not affected by participation or withdrawal from the project. Participants are allowed sufficient time (5 days minimum) to consider the information, before the researcher returns to answer any questions the participant may have and note whether the individual wishes to participate and give consent for participation. A prompt card, highlighting key facts about the research is provided to the nursing, occupational therapy and care staff who will assist with identifying participants based on the inclusion criteria. Efforts are made to ensure that family members/carers are informed of the study. All participants are fully debriefed and given a contact for any further questions or for making any complaints.

In site 1, care homes are identified through the partnership with Equal Arts (the community arts partner), and initial meetings are arranged to explain the study to the manager, and then with their approval the residents and staff. In site 2, participants are identified and recruited in partnership with Derbyshire Community Services NHS Trust (DCHS). There are three wards/units across two hospitals who all admit people with dementia. The main points of contact for these wards will be through the head occupational therapist and ward manager. In site 3, people living in the community are recruited through NHS general practices in the area, memory clinics and local clubs that provide services for people living with dementia.

##### Inclusion criteria

- Diagnosis of dementia or evidence of age-related memory impairment.
- In Newcastle/Tyne and Wear—participants will be a resident in the chosen care home.
- In Derbyshire—participants will be a resident in the assessment unit for a minimum of 3 months.
- In North Wales—participants will be living in the community in rented/private housing or sheltered housing.

##### Exclusion criteria

- Recent or current episode of major mental illness.
- End of life/terminal illness.
- Debilitating illness that will preclude regular attendance.
- Severe uncorrected sensory or communication difficulty.
- Inability to communicate verbally through the medium of either English or Welsh.

##### Assessing capacity and gaining informed consent

The Mental Capacity Act (MCA; 2005) states that a person must be assumed to have capacity unless it is established otherwise. To establish whether the potential participant's memory difficulties may impact on the capacity make an informed choice, capacity will be assessed by the researcher at the point it needs to be made (ie, when discussing the study with a view to gaining consent). The researcher will assess (1) the capacity to understand the information relevant to the decision to be made, (2) retain the information, (3) use or weigh the information to arrive at a choice/decision, and (4) ability to communicate a decision. In line with the MCA Code of Practice,^30^ the first three assessments are applied together. If a person cannot do any of these three things, they will be treated as unable to make the decision. The fourth only applies in situations where people cannot communicate their decision. If there is an indication of a lack of capacity, and a person is unable to give informed consent, an opinion is sought from a family member/carer/suitable person to act as a personal consultee and provide consent. Consent is regarded as a continuing process rather than a one-off decision, and willingness to continue participating will be continually checked through discussion with participants during the assessments.

##### Data collection (people living with dementia)

The following quantitative and qualitative questionnaire data are collected in a face-to-face interview by the researcher at time 1 (baseline) prior to the start of the intervention, time 2 (follow-up) at the end of the intervention and time 3 (longer term follow-up) 3 months after the time 2 assessment.

- Demographic data (eg, age, gender, marital status, socioeconomic status, education, residence).
- Social connectedness (Lubben Social Network Scale, six items;^31^ emotional and social loneliness, six items).^32^
- Dementia Quality of Life.^33^ A 28-item measure of QoL designed for people with dementia.
- Medication use (limited to particular types of psychotropics and those prescribed for dementia).
- Extent of engagement with art activities (qualitative).
- Expectations of taking part (qualitative, baseline only).
- Sense of belonging to community (five items).
- Memory of the sessions (qualitative, follow-up only).
- Perceived benefits and value (qualitative, follow-up only).

##### Associated measures for people living with dementia

- Clinical Dementia Rating Scale:^34^ administered at T1 only, to establish severity of the symptoms of dementia, completed by researcher.
- Dementia Quality of Life:^33^ a 31-item measure of QoL designed for carers of people with dementia (to be completed by carer or staff).
- Holden Communication Scale:^35^ assesses a range of social, behaviour and communication variables (to be completed through discussion with carer or staff member).

##### Behavioural observation of people living with dementia

A within-participants/repeated measures 2×3 design will compare levels of well-being and engagement in two different conditions (intervention and control). The control condition is an unstructured activity. Using the Greater Cincinnati Chapter Well Being Observational Tool,^18^ observations are coded by the researcher according to the extent to which well-being and engagement are expressed at three time points: time 1, baseline (control condition only); time 2, at the start/within the first 2 weeks of intervention; time 3, the last 2 weeks of intervention. The Greater Cincinatti Chapter Well-Being Tool addresses seven domains of well-being—interest, sustained attention, pleasure, negative affect, sadness, self-esteem, and normalcy—and was developed specifically to observe the effects of visual arts on the well-being of people with dementia.

The aim of the observations in the control situations is to ensure a concurrent control for the person's function and interaction in the arts sessions, bearing in mind there may be changes over time unrelated to the project intervention. On that basis, we will undertake observations between situations within a few days of each other. Observations in each time point are carried out over 2 weeks to account for variability between sessions, as suggested by Brooker and Duce.^36^

This systematic method of observation enables an examination of the unconscious responses, that is, those that might not be verbalised during interviews. Where possible, the sessions are videoed to enable further analysis and validation. Care will be taken to ensure that others present who are not research participants cannot be identified in the filming. The videos are purely for research purposes and none of their content will be made public or shared beyond those involved in the analysis.

##### Focus groups with people living with dementia

In each of the data collection areas a short, postsession focus group is held in the last month of the intervention period with each of the intervention groups (up to 4 in each area, 12 in total). This will only be for the participants with dementia, to ensure that any effects that are experienced ‘in the moment’ are verbalised from their own perspectives. This session is led by the researcher and will be videoed or audio recorded for research purposes only, the video/audio recording is not to be made public or shared beyond those involved in the analysis.

##### Self-reported outcomes from people living with dementia

For those people living with dementia who are able, a short, five-item questionnaire developed by the research team is completed immediately after the end of an arts session. Each question, for example, ‘How interesting was the session?’ has a visual ‘smiley faces’ response scale, with associated scores ranging from ‘1=not great’ (sad face) through to ‘10=fantastic’ (very happy face). These will be collected at the beginning (weeks 1 and 2) and the end (weeks 11 and 12) of the 12-week intervention. This will ascertain individual ‘in the moment’ responses on interest, enjoyment, feelings, friendliness, achievement.

### Analysis of study sample 1

#### Primary outcomes

Change over time (T1 and T2 and longer term T3) for significant improvements in the Dementia Quality of Life Scale (DEMQOL) and Holden Communication Scale will be ascertained using a repeated measures mixed-effects model. It will examine the extent to which the number of sessions attended, location, age, gender, education, socioeconomic status, medication use, Clinical Dementia Rating Scale (CDR) score and location are associated with outcomes. Differences between the three settings will be tested. Should these not be significant, data will be combined to increase sample size. Change over time in observed well-being between times 1 and 2, and differences between the control and intervention will be examined with a repeated measures mixed-effect model.

#### Secondary outcomes—suggestions

A process analysis will examine how changes in outcomes may be explained by other factors. Controlling for demographic factors, mediation analyses will examine if changes in well-being and QoL are influenced by memory/recall of the art group, if improvements in social connections explain changes in QoL and well-being, and if recent arts engagement is associated with better outcomes.

The focus group data and open-ended questions from the questionnaire will be subject to thematic analysis^37^ This will explore the participants' views on what they liked and disliked, their feelings about the impact of taking part and social interactions. The self-report data will be integrated and analysed with the focus group and observation data, to enable a deeper understanding of the impact of the intervention.

### Study sample 2: staff and carers

#### Recruitment procedures

Across the three sites, the staff or carers are recruited as per the participants living with dementia. In sites 1 and 2, we will approach a wide range of professional care staff (eg, managerial, nursing, occupational therapists, care support workers, activity coordinators). In site 3, we will recruit participants who are the spouse, family member or friend of the participant with dementia. This involves three stages to gaining consent: (1) the researchers visit/promote and explain study; (2) potential participants express interest and receive information sheet, appointment booked to revisit (with sufficient cooling off period of 5 days); and (3) researchers meets with participant, explains study, answers questions, assesses capacity and obtains consent for participation.

##### Inclusion criteria

- Member of staff in residential care homes or NHS facility, or spouse, family member or friend of the participant living with dementia.

##### Exclusion criteria

- Recent or current episode of major mental illness.
- End of life/terminal illness.
- Inability to communicate verbally through the medium of either English or Welsh.

##### Data collection

The following quantitative and qualitative data are collected in a face-to-face interview with the carer or member of staff by the researcher at time 1 (baseline) prior to the start of the intervention, time 2 (follow-up) at the end of the intervention and time 3 (longer term follow-up) 3 months after the time 2 assessment.

- Demographic data (eg, age, gender, marital status, post held).
- Self-reported health.
- Proximity to participant in relation to the intervention.
- Attitudes towards dementia—the Approaches to Dementia Questionnaire.^38^ A 19-item scale that assesses a person's attitudes to dementia, including subscales of ‘person-centred’ and ‘hope’.
- Perceptions of arts activities, and changes over time (single-item/open-ended questions).
- Changes in their friend/client/family member living with dementia (T3 only).

### Analysis of study sample 2

#### Primary outcomes

Change over time (T1 and T2 and longer term T3) in the Approaches to Dementia Questionnaire will be examined with a repeated measures mixed-effects model, and the extent to which proximity to the participant in the intervention influences attitudes.

#### Secondary outcomes—suggestions

This will examine the types of changes (if any) that are reported in their friend/client/family member and the relationships between these and attitudes towards dementia.

### Study sample 3: art practitioners and other staff/professionals

To examine contagion effects, across all sites up to nine arts practitioners (and their organisations) involved in the intervention will be invited to take part. Up to nine participants in the geographic locations in which the interventions are delivered (eg, residential care homes, art galleries, NHS assessment unit, villages) will be purposively selected on the basis of a high level of engagement with the project compared with others in the locations, for example, care staff attending art sessions. This will involve an electronic survey, collecting the information described in table 1.

**Table 1:** Data collected from art practitioners, staff/professionals

Art practitioners	Staff/professionals
Demographic data, including *professional age* measures how long the artist has been practising with people with dementia. *Number of journals* an artist receives or subscribes to as a measure of media exposure; *number of conferences attended in the last year as* a measure of network exposure; artists with *an honorary position* in their hospital/local authority; *artistic orientation*, coded as ‘1’ if the artist agrees with the statement that it is more important for an artist to ‘keep herself/himself informed of new artistic interventions than to devote time to practising art’ and ‘0’ otherwise.	Demographic data, including attitudes, beliefs and knowledge about people with dementia (Approaches to Dementia Questionnaire)^38^

Discussion and collaboration network data (adapted from Coleman *et al*,^39^ and Kahn and Antonucci,^40^ to be developed by researcher).

The perceived benefits of the intervention.

Self-reported leadership (adapted from Childers,^41^ to be developed by researcher).

Further waves of data collection will take place with (1) artists and (2) other professionals, for example, care staff, identified through the discussion and collaboration networks.

### Analysis of study sample 3

Indegree centrality scores are calculated for both samples. This is the number of other artists/practitioners who nominate or ‘send network ties to’ a particular artist/practitioner, and likewise the number of other colleague/relative/friends who nominate or ‘send network ties’ to other relatives/friends. Indegree centrality is computed for each person separately in the collaboration, discussion and total network (in the latter, indegree is the sum of the indegree in the collaboration and discussion networks). Indegree centrality is the most basic measure of status or prestige in a network. Since we will measure the social ties as pertaining to collaboration and discussion of the intervention with others for artists, those with greater indegree centrality are both ‘social connectors’ and recognised ‘experts’. Using these data, we will document how sociometric leadership, self-perceived leadership inter-relate in affecting social contagion effects concerning the intervention, and beliefs about dementia.

### Qualitative semistructured interviews with subsamples of people living with dementia and carers/staff

#### Data collection

In-depth semistructured interviews will be conducted with a (1) subsample of participants living with dementia (n=35) and (2) their (informal or formal) carers (n=35) across the same time frame as the quantitative data collection, during an additional visit to the participant. The selection is based on the availability of a dyad (person living with dementia and a carer) to examine convergence/divergence in beliefs over the course of the study. Interview topic guides will explore:

- The participants' previous engagement with art, using cultural capital^ii^ as a theoretical framework to guide the analysis. This explores attitudes towards art, what people define as ‘art’, engagement with ‘art’ in galleries or everyday art. This will enable changes in arts engagement to be understood. The construct of social capital^iii^ is used to aid the analysis of connectivity, as it provides a systematic way of thinking about the qualities of communities (baseline).
- The boundary and nature of the community as experienced by the people with dementia themselves, exploring how community boundaries are perceived, the extent to which opportunities exist to interact with others within the community boundary and how this changes over the time of the intervention (baseline).
- The changes in the above themes (a and b) and the extent to which changes (if any) have been sustained and/or lead to new aspects of social connectivity (T2 and T3).

#### Analysis

Qualitative data will be digitally recorded and professionally transcribed for analysis. Software such as QSR NVivo will be used to aid analysis. Thematic analysis^37^ is used to identify themes that originate from the data (inductive) and the analysis may also be guided by the theory (deductive), such as community and in terms of responses to art, cultural and social capital. Detailed readings of the interview transcripts will help establish a familiarity with the data and encapsulate the emerging themes. An outline coding scheme to highlight meaning will be developed.^43^ The range and variation of themes may be explored using data displays.^44^

### Health economics

The health economics component conducts a social return on investment (SROI) evaluation of the intervention. This involves six stages:^45^ establishing the scope and identifying key stakeholders; mapping outcomes; evidencing outcomes and giving them a value; establishing impact; calculating SROI; reporting, using and embedding. The principles are: stakeholder involvement; understanding changes; valuing things that matter; including only what is material; avoidance of overclaiming; transparency; verification of results. A small number (up to 15) separate interviews will be held with stakeholders directly involved in the study and with those who work in the area but are not directly involved in the study. Questions included in the main interviews focus on the need to establish value ‘contingent valuation’ (which can be monetarised) and in determining impact.

#### Analysis

The evaluative SROI will be conducted in accordance with the principles and methodology set out in the Cabinet Office guide.^45^ This involves (1) mapping outcomes, constructing an impact map informed by the engagement with stakeholders. The range of costs and benefits to be measured will be identified; (2) evidencing outcomes and giving them a value; (3) establishing impact, considering deadweight and displacement (what would have happened without the intervention), attribution (how much of the outcome should be assigned to the activities of other organisations), drop-off (how long the outcomes lasted); (4) calculating the SROI, using the data assimilated in the earlier stages to calculate the SROI and conduct appropriate sensitivity analysis; (5) reporting, using and embedding.

### Public engagement

Public engagement projects using the work from Dementia and Imagination are under development. These will be highly interactive with a national, strategic focus and regional activity. An example of the activities is given below in table 2.

**Table 2:** Example of public engagement activities

Activity/project title	Engagement questions	Outcomes/output	Target of activity	Data
Practising imagination through visual art	How can I have fun with my clients/spouse/partner? How can I learn more about helping someone living with dementia? How can I learn what a person with dementia is capable of? What are the best ways of using visual arts to enrich the lives of those with dementia?	Each of the 3 sites to run practitioner workshops/training sessions, using ‘A Practitioners Guide to Dementia and Imagination’ and online resources	More people delivering arts activities as part of care for people living with dementia Developing skills for those working in the care of people living with dementia, including family carers	Short evaluations; longer term follow-up to ascertain practice changes

### Artistic practices

Most interventions using artistic and cultural methods are assessed primarily with regard to their impact on participant individuals or communities. There has been much less interest in the specific cultural or aesthetic element of interventions or the impact on the aesthetic or artistic practitioners themselves. This is developed in three ways, using longitudinal group interviews with three artists employed to respond, aesthetically, to the research programme; using focus groups with the artistic practitioners delivering the interventions; and exploring participatory and coproduced methods over the course of the project. These approaches aim to answer the core question(s) of how the practitioners' understandings of dementia change in response to the project, how the practice itself is affected (including, eg, the use of new or mixed aesthetic methods), and the practitioners relationship to research. These three questions contribute to the gap in evaluation literature that has, to date, not fully engaged with the aesthetic practice aspects of aesthetic interventions.

### Data integration across the work programme

Results from the quantitative and qualitative data for all work packages will be integrated as much as possible. There are various ways of combining the results from qualitative and quantitative data sets, in addition to corroboration, which will inform the synthesis of our data.^46^ These include elaboration, where one type of data adds to the understanding being gained by another type of data; complementarity, where analysis of qualitative and quantitative data sets generate complementary insights that improve overall understanding of a particular topic; and contradiction, where qualitative and quantitative findings conflict and give rise to juxtapose positions to be explored in further research. The synthesis of quantitative and qualitative data will ensure that the findings are as comprehensive as possible and provide a sound basis for generating research, policy and practice recommendations. Data integration will enable further theoretical development in visual art and dementia research. The longitudinal element of the concurrent data collection provides an opportunity to examine the temporal aspects.

### Ethics and dissemination

All participant information provided is prepared in an acceptable manner that is clear and understandable. Bilingual information (Welsh and English) is prepared in Wales. All research staff and artists who will have direct contact with participants will undertake a programme of training developed by the research team. This will cover the MCA, informed consent, dementia awareness and the study protocol procedures, including data collection methods and data management. Professional standards as set out in the Research Councils UK (RCUK) Policy and Guidelines on Governance of Good Research Conduct are adopted by the study.

A requirement of UK research councils is that data resulting from the study are shared with the wider research community through the UK Data Archive. Data management protocols will be followed to ensure confidentiality and anonymity and protection of personal data. All data will be anonymised and stored separately from any identifying details. For contact purposes, these are linked through a unique identifier. Hard copy data will be stored securely in locked filing cabinets and electronically in password-protected folders. Access will only be available to the research team. The lead investigator will coordinate the sharing of data within the research team. Any transmission of data/transcripts will be through a secure method and encrypted. In terms of digital data (film/photography/audio) all qualitative interviews will be recorded using digital recording devices and securely stored as audio files (mp3) and encrypted.

Two types of visual data will be obtained. One is through videoing the observation sessions, and is purely for research purposes only. This will be securely stored through networked security. The other is artistic output, which will be handled sensitively, with the wishes of the participant being observed at all times. This will be securely stored and its use managed through the research team. Copyright will be held with specific investigators in the research team at each of the three data collection sites (as they are the employers).

The research findings will be shared through a range of activities. International and national academic conferences and events will be attended to present papers and lead symposia. The project has developed an extensive public engagement and communication strategy to maximise the planned pathways to impact. This takes into account the variation in needs, concerns and preferred ways of working and communicating of each significant beneficiary group. Public engagement projects will target a broad range of stakeholders. A project website will be developed as a resource bank for stakeholders and a continuing legacy from the project. A quarterly newsletter will be produced. Policy and practice summaries will be developed from the findings. The current visual arts intervention protocol will be developed as a practitioners guide, freely available as an electronic resource as well as a hard copy version.

## Discussion

Dementia and Imagination is the largest arts and dementia research study in the UK. It seeks to address not only health-related issues, but broader societal factors that impact on the lives of people living dementia, notably the right to social inclusion, good quality care and supportive communities. Drawings on a range of scientific areas are highlighted as an important contribution to future dementia research.^47^ The combination of arts and science in this research enables the generation of a broad range of data for an in-depth synthesis. Despite the strengths of the study, some limitations should be recognised at the outset. A major challenge for dementia research is measuring the impact of psychosocial interventions. A number of standardised and validated measures exist, some we incorporate into our protocol. However, the extent the measures are sensitive enough to capture change in relation to a complex intervention such as a visual arts intervention are unclear. In recognising these challenges at the outset, the mixed-methods approach will enable qualitative exploration which may reveal more subtle impact and experiences to be identified.

The economic analysis adopts a SROI, which uses a broader stakeholder perspective than a cost-utility analysis, but it is open to some criticism.^48^ ^49^ One of the key criticisms is that while SROI evaluations typically report detailed information on sources of information and financial proxies, the arbitrary nature of selecting financial proxies is an area for concern as much depends on the assumptions made by the researchers. To minimise the risk of overclaiming benefits, SROI analyses incorporate deadweight, attribution and drop-off rates. In Dementia and Imagination, we include questions about participants' other activities to obtain more robust estimates of deadweight and attribution, and the drop-off will be calculated by observing differences between baseline and end of study values.

A further limitation is that the study design cannot focus on a more robust test of effectiveness, as a randomised controlled trial was beyond the remit of the available resources. However, with the exception of randomisation, the study assesses a range of processes that would traditionally be the focus of a pilot study (eg, recruitment, retention, eligibility criteria, different forms of data collection with a range of measures, personnel issues, data management, etc) which will usefully serve the next stage in seeking funding for a definitive trial of the intervention.

## Footnotes

i Based on a qualitative analysis of N=28 people with mild-to-moderate dementia taking part in a museum-based activity.

ii Cultural capital is defined by Bourdieu^42^ as (1) embodied state, “in the form of long-lasting dispositions of the mind and body” (p. 47); (2) an “objectified state, in the form of cultural goods” (p. 47); and (3) an institutionalised state such as educational qualifications.

iii Social capital is defined by Bourdieu^42^as the “aggregate of the actual or potential resources which are linked to possession of a durable network of more or less institutionalised relationships” (p. 51).
