# Supplementary material for: Dementia and Imagination: a mixed-methods protocol for arts and science research
Source: BMJ Open. 2016 Nov 2;6(11):e011634. doi: 10.1136/bmjopen-2016-011634 (PMC5129039; doi:10.1136/bmjopen-2016-011634)
Supplement: supplementary appendix [file bmjopen-2016-011634supp_appendix.pdf]

Appendix 1: Dementia and Imagination Work-Packages

	**WP1: Understanding the impact of visual arts on well-being and quality of life**
Title:	*Part1 –Development of visual arts programme*
Objective:	Production of a theoretically informed visual arts intervention for people living with dementia
Led by:	Gill Windle and Clive Parkinson
Title:	*Part 2 - Integration:*
Objective:	To integrate the newly produced evidence from each of the other WPs to generate theoretically informed holistic conclusions about visual arts interventions, and the implications for research, policy and practice on the value of arts research.
Led by:	Gill Windle and Dave O'Brien
	**WP2: The role of art**
Title:	*Part 1: Art in the research process*
Objective:	To generate output to ensure the research findings will be more widely understood.
Led by:	Clive Parkinson and Dave O'Brien
Title:	*Part 2: Engagement through art*
Led by:	Victoria Tischler
Objectives:	To raise awareness of dementia and challenge attitudes. Sharing work in a range of non-traditional settings will provide greater opportunities for dissemination and public response from diverse communities.
	**WP3: Community connectivity through visual arts** In order to establish whether the visual arts intervention improves community connectivity and well-being, this work package is structured in three parts, over specific periods of time. Baseline (T1), follow up (T2) and long term follow up (T3)
Title:	*The impact of the programme on the connectivity and well-being of people with dementia*
Led by:	Gill Windle and Bob Woods
Objective:	To respond to the identified need for further research to capture the subjective, unique aspects of the experience and meaning of the programme on the well-being of people with dementia (Mental Health Foundation, 2011); compare

	changes over time in well-being, communication, quality of life, memory, use of medications, receipt of services and self reported health; compare the intervention against a control condition.
Title:	*Changes in social connectedness of people with dementia*
Led by:	Ness Burholt and Andrew Newman
Objectives	To ascertain quantitative changes in social resources and loneliness.<br><br>To understand qualitative changes in connectivity and communication at the micro-level between the participant and formal or informal carer(s) and other network members.
Title:	*Social Contagion: Changes in the community of practice*
Led by:	Ness Burholt
Objectives	To investigate contagion dynamics over social networks in a real community of practice which will be of relevance to both practitioners and researchers (Van den Bulte & Lilien 2001; Watts & Peretti 2007).
Title:	*Social contagion: Changes within the community of place*
Led by:	Ness Burholt
Objectives	To investigate contagion dynamics in the wider communities of place, which are the geographic locations in which the interventions are delivered (e.g. residential care homes, art galleries, NHS assessment unit, villages). This analysis is based the same three underlying principles outlined above in the work with practitioners.
Title:	**WP4: Understanding the economic value**
Objectives:	To develop an economic case for socially engaged visual arts interventions, adopting a variation of cost benefit analysis, social return on investment (SROI), to measure the social environmental and economic costs and benefits.
Led by:	Rhiannon Tudor-Edwards with Carys Jones
Title:	**WP5 Maximizing engagement and research impact**
Objectives:	To facilitate the impact of the research, knowledge exchange with stakeholders, partners, policy and interested organisations, and engagement with our research communities.
Led by:	Michael Baber
